# A randomized clinical trial investigating the impact of magnesium supplementation on clinical and biochemical measures in COVID-19 patients

**DOI:** 10.1186/s12985-024-02362-6

**Published:** 2024-04-23

**Authors:** Sepideh Rostami, Seyed Mohammad Alavi, Robab Daghagheleh, Elham Maraghi, Seyed Ahmad Hosseini

**Affiliations:** 1 Department of Nutrition, School of Allied Medical Sciences, Ahvaz Jundishapur University of Medical Sciences, Ahvaz, Iran; 2 Jundishapur Infectious and Tropical Diseases Research Center, Ahvaz Jundishapur University of Medical Sciences, Ahvaz, Iran; 3 Department of Biostatistics and Epidemiology, Public Health Faculty, Ahvaz Jundishapur University of Medical Sciences, Ahvaz, Iran; 4 Nutrition and Metabolic Disease Research Center, Ahvaz Jundishapur University of Medical Sciences, Ahvaz, Iran

**Keywords:** COVID-19, Magnesium, Dietary supplements, Clinical trials, Disease management

## Abstract

**Background:**

This study sought to examine the impact of magnesium supplementation on clinical outcomes and biochemical factors among hospitalized patients with COVID-19.

**Methods:**

This double-blind, randomized clinical trial was conducted at Razi Hospital, Ahvaz, Iran, between September 2021 and March 2022. Participants aged 18–70 years with moderate disease severity were enrolled. Magnesium supplementation (300 mg daily) was administered to the intervention group, while the control group received a placebo. Clinical outcomes, including the need for oxygen therapy, oxygen saturation, respiratory rate, fever, hs-CRP and TNF-α levels, as well as quality of life and mental health, were assessed. Blood samples were collected to measure biochemical variables.

**Results:**

The main result was the count of individuals requiring oxygen therapy. Additional outcomes comprised of oxygen saturation, respiratory rate, fever, hs-CRP and TNF-α levels, as well as quality of life and mental health. Out of 64 participants, 60 completed the study. The results showed that magnesium supplementation significantly reduced the number of patients requiring oxygen therapy (9 vs. 14; *P* < 0.001). Moreover, the magnesium group demonstrated improved oxygen saturation compared to the control group (4.55 ± 2.35 vs. 1.8 ± 1.67; *P* < 0.001). Furthermore, we observed a noteworthy enhancement in the quality of life and depression score in the magnesium group. No significant differences were observed in respiratory rate, fever, hs-CRP, and TNF-α levels (*P* > 0.05).

**Conclusion:**

The findings suggest that magnesium supplementation may have beneficial effects on clinical outcomes and arterial oxygen saturation in COVID-19 patients. More investigation is necessary to delve into its potential mechanisms and long-term effects on patient outcomes.

**Trial registration:**

This study is registered on Iranian Registry of Clinical Trials (IRCT) under identifier IRCT20210413050957N1. (The registration date: May 1, 2021).

## Introduction

The global outbreak of COVID-19 caused by the SARS-CoV-2 virus has presented an unprecedented challenge to public health worldwide, prompting an urgent search for effective treatment strategies [1]. As of March 19, 2023, the worldwide consequences of the COVID-19 pandemic triggered by the SARS-CoV-2 virus led to in 761,402,282 confirmed cases and 6,887,000 fatalities worldwide [2]. Since 2019, this disease has become the biggest health-related challenge in the whole world and has imposed a lot of financial problems on healthcare systems [3].

The human body relies on its immune system as the primary defense mechanism against viral infections and diseases [4, 5]. Upon pathogen invasion, various immune response pathways are activated to combat the threat [6, 7]. Nutrition plays a pivotal role in enhancing these immune response mechanisms, thereby bolstering the body’s ability to defend against pathogens [8–10]. Studies have demonstrated a notable increase in the risk of mortality and adverse events during hospitalization among adult COVID-19 patients with nutritional deficiencies or malnutrition [11–13]. In COVID-19 patients, malnutrition or susceptibility to malnutrition correlates with heightened inflammation and compromised immune function, potentially exacerbating disease progression and complicating its outcomes [14, 15]. Hence, nutrients with the potential to enhance immunity against the coronavirus (COVID-19) and mitigate the risk of infection or progression of associated factors play a crucial role in both preventing the disease and treating afflicted individuals [16, 17].

Among the various micronutrients under investigation, magnesium has gained attention due to its essential role in immune function, pulmonary health, and cellular metabolism [10, 18]. Magnesium (Mg) is the second most plentiful positively charged ion found within cells, following potassium. About 99% of the total magnesium in the body is concentrated within the intracellular area, while approximately 1% is distributed in the bloodstream and other fluids outside the cells [19–21]. Low levels of magnesium in the blood, which are quite prevalent in some countries, are commonly observed among elderly individuals due to inadequate dietary intake, certain health conditions (such as diabetes), and the use of multiple medications [12, 22, 23].

Mg partakes in various metabolic and biochemical processes and plays a crucial role in numerous essential functions in the body. These include bone development, neuromuscular activity, signal transmission, energy production, as well as the metabolism of glucose, lipids, and proteins [24, 25]. Additionally, Mg contributes to the stability of DNA and RNA, as well as the regulation of cell growth and specialization [26]. It’s important to mention that magnesium (Mg) plays a role in controlling the functions of both the innate and adaptive immune systems [27]. This can potentially provide protective benefits against COVID-19. For example, Mg helps stabilize mastocyte membranes, regulates the activity of neutrophils and macrophages, and hinders the Toll-like receptor a/nuclear factor-κB (NF-κB) pathway [28, 29]. Moreover, magnesium (Mg) adjusts the cytotoxic activities of natural killer (NK) cells and CD8 + T lymphocytes [30].

Given the significance of magnesium in immune modulation and its prevalent deficiency among patients, this study aimed to investigate the impact of magnesium supplementation on clinical outcomes and biochemical factors in hospitalized COVID-19 patients. By addressing these critical gaps in the literature and providing novel insights into the potential benefits of magnesium supplementation, this study contributes to the ongoing efforts to optimize treatment strategies for COVID-19.

## Methods

### Research design and participant enrollment

This research conducted at Razi Hospital, Ahvaz, Iran, between September 2021 and March 2022, involved a double-blind, randomized clinical trial on COVID-19-infected patients who were hospitalized in the infectious ward. In order to qualify for involvement, people were required to fulfill the following conditions: be aged between 18 and 70 years, have received a verified COVID-19 diagnosis via a positive RT-PCR test, nasopharyngeal swab, experiencing respiratory symptoms (such as breathlessness, chest discomfort, and sensations of pressure) with or without an accompanying fever (equal or more than 38 °C) and Spo2 between 90% and 93%. Patients with moderate disease severity were included in this study. Patients who were critically ill and were hospitalized in the intensive care unit (ICU) or had a mild illness and did not need to be hospitalized were not included in the study.

Other inclusion criteria include absence of pregnancy and breastfeeding, without history of diabetes, high blood pressure, heart disease, kidney dysfunction, and psychological disorders like depression, Body mass index less than 35 and more than 18, Vitamin D level above 30 ng/ml, absence of hypomagnesemia that refers to a deficiency in magnesium levels (below 1.7 mg/dL) and hypermagnesemia signifies an excess of magnesium levels (above 2.6 mg/dL), and absence of alcohol and drug abuse. Exclusion criteria included unwillingness to continue participating in the study, hospitalization of the patient in the ICU, Taking antioxidant and anti-inflammatory food supplements such as vitamin E, omega 3, vitamin D, etc. and contraindication, intolerance or allergy to magnesium supplement.

The sample size calculation was based on a previous study by Tan et al. [31] which reported a 60% reduction in the proportion of cases requiring oxygen therapy in the intervention group compared to the control group. With a power of 80%, alpha level of 0.05, and estimated proportions of 61.5% in the control group and 24% in the intervention group, the sample size was determined to be 25 individuals per group. Considering a 20% attrition rate, the final sample size comprised 30 individuals in each group.

$${\rm{n}}\,{\rm{ = }}\,{{{{\left( {{{\rm{Z}}_{\left( {{\rm{1}} - {\raise0.7ex\hbox{${\rm{\alpha }}$} \!\mathord{\left/{\vphantom {{\rm{\alpha }} {\rm{2}}}}\right.\kern-\nulldelimiterspace}\!\lower0.7ex\hbox{${\rm{2}}$}}} \right)}}\,{\rm{ + }}\,{{\rm{Z}}_{\left( {{\rm{1}} - {\rm{\beta }}} \right)}}} \right)}^2}\left[ {{{\rm{p}}_{\rm{1}}}\left( {{\rm{1}} - {{\rm{p}}_{\rm{1}}}} \right)\,{\rm{ + }}\,{{\rm{p}}_{\rm{2}}}\left( {{\rm{1}} - {{\rm{p}}_{\rm{2}}}} \right)} \right]} \over {{{{\rm{(}}{{\rm{\varepsilon }}_{{\rm{12}}}}{\rm{)}}}^{\rm{2}}}}}$$

**Fig. 1:**
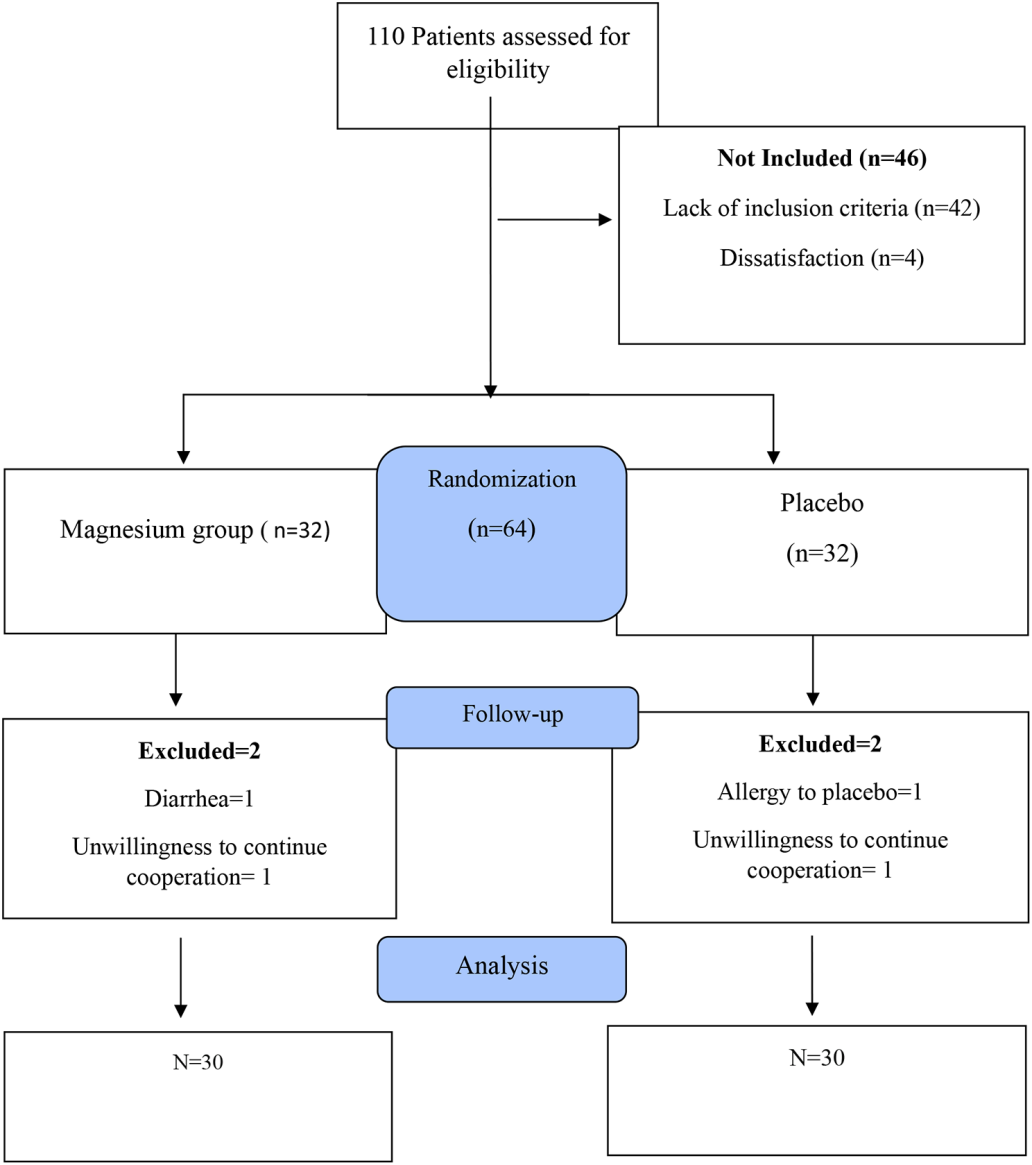
Flowchart of the study protocol

The general scheme of the intervention is shown in Fig. 1. The method of assigning patients to the intervention and control groups was the approach of employing randomized permutation blocks with a block size of 4 (referring to the table associated with random permutations). The participants were randomly allocated in a ratio of 1:1 to the intervention and control group. A computer-generated list was created using random permuted blocks to ensure that the allocation to subjects and investigators remained concealed. The intervention and placebo were kept hidden from researchers, patients, infectious disease specialist and clients. Magnesium supplements and placebo were completely similar in terms of appearance, color, fragrance, and packaging, so that the blinding process could be fully implemented.

At the study’s inception, the researchers thoroughly briefed the patients on the process of implementing the study and all participants provided written informed consent to partake in the study. Patients had the option to exit the study at any point if they were unwilling to cooperate. The study was conducted in compliance with the Declaration of Helsinki and the protocol of the research was approved by the ethics committee of Ahvaz Jundishapur University of Medical Sciences (Ethical code: IR.AJUMS.REC.1400.0255) and This study is registered on Iranian Registry of Clinical Trials (IRCT) under identifier IRCT20210413050957N1. (The registration date: May 1, 2021).

### Intervention

Participants within the intervention group were provided with 300 mg of oral magnesium supplement in the form of magnesium citrate capsules daily, and subjects in the control group received a placebo containing starch. Magnesium citrate powder was obtained from Behan Sar Pharmaceutical Company, and capsules containing magnesium and placebo were prepared by an expert at the Faculty of Pharmacy of Jundishapur University and provided to the study. The placebo capsules contained starch and were similar in appearance to magnesium supplements. The duration of intervention in patients was from the time of admission to the time of discharge from the hospital. In case of changes in the diet and medication protocol of the patients, the changes were reviewed by the research team, and in case of interference with the study objectives, the patient was excluded from the study.

### Data about the physical dimensions of the body and past medical records

Information pertaining to age, weight, height, past medical conditions, prescribed medications, blood pressure, serum lipids, random blood glucose, and respiratory condition was obtained by examining medical records.

### Dietary intake

Nutritional intake of patients was done by recording of 24-hour dietary recall at both the start and conclusion of the research. The N4 software was utilized to analyze data on dietary intakes, with national food composition tables serving as a reference for the analysis of food intake.

### Primary and secondary outcome

The confirmatory result of this study was the number of patients who needed oxygen therapy. Secondary outcomes included SaO2, respiratory rate, fever, hs-CRP and TNF-α levels, the well-being and psychological state of individuals’ lives and mental health.

### Measurements

To measure biochemical variables, 7 cc of blood were taken from the patients at the beginning of the study and on the day of discharge from the hospital. Diaclone Research’s ELISA kits from Besançon, France were employed to evaluate the levels of hs-CRP and TNF-α concentration. To assess serum magnesium concentration, the measurements were conducted using an auto-analyzer utilizing a photometric method (BT-3500, Biotecnica Instruments, Rome, Italy) using Biosystem Kits (Barcelona, Spain). Depression status of patients was measured by Beck Depression inventory-II (BDI-II). The research conducted by Hamidi and colleagues, Persian version of the questionnaire was utilized, demonstrating satisfactory validity (alpha = 0.92) and reliability (*r* = 0.64) [32]. Individuals participating in the study filled out a self-report inventory consisting of 21 items. The items were rated on a scale of 0–3, where a higher score indicates more severe symptoms. The total score of the questionnaire is then classified into the following categories [33]: for scores ranging from 14 to 19, it indicates a mild level of depression. Scores between 20 and 28 suggest a moderate level of depression, while scores between 29 and 63 indicate a severe level of depression. The patients’ anxiety was evaluated by Spielberger State-Trait Anxiety Inventory (STAI). In Abdoli et al.‘s study, the Persian version of the questionnaire was employed, indicating acceptable validity (alpha = 0.88) and reliability (*r* = 0.64) specifically for measuring state anxiety [34].

The researchers employed the SF-36 questionnaire to evaluate the quality of life of individuals in this study. The SF-36 is a frequently employed survey that individuals fill out on their own, providing a comprehensive assessment of their quality of life related to health. It consists of 36 items grouped into eight subscales, which are further classified into two broad components: the physical component summary (PCS) and the mental component summary (MCS). The physical component summary comprises evaluations of 10 items related to physical functioning, 4 items related to role-physical, 2 items related to bodily pain, and 5 items related to general health perceptions. The mental component summary comprises the following subscales: social functioning (2 items), role-emotional (3 items), mental health (5 items), and vitality (4 items). Additionally, there is a single item that separately assesses changes in health. The scores for all items are assigned numerical codes, summed together, and then transformed into a scale ranging from 0 to 100. In this scale, a score of 0 represents the poorest condition, while a score of 100 indicates the best condition [35]. In 2006, Montazeri et al. conducted a study in Tehran, Iran, to validate the SF-36 questionnaire among a healthy population aged 15 years and above. The study confirmed the reliability of the questionnaire by calculating Cronbach’s alpha coefficients, which ranged from 0.77 to 0.90 for the subscales and the overall scale [36].

### Statistical analyses

The quantitative data were expressed as the mean value accompanied by the standard deviation. On the other hand, qualitative data was presented in the form of percentages and numerical counts to describe the status of these measurements. The Kolmogorov-Smirnov test was employed to validate the normal distribution. The study employed the Independent T-test and chi-square test to evaluate and draw comparisons between groups’ results at the beginning and conclusion of the study. Changes from the initial baseline were analyzed within each group using a paired samples t-test. The analysis of covariance (ANCOVA) test was used to account for confounding factors. All ANCOVA models were adjusted for weight, serum magnesium levels at baseline, and calorie intake. The data underwent analysis with SPSS19 software, and if the P-value was less than 0.05, it was deemed statistically significant.

## Results

### Patients

Out of the 110 patients evaluated for qualification, 64 met the criteria and were randomly assigned to either the magnesium group or the placebo group. Some participants couldn’t be included because of the following reasons: pregnancy, taking antioxidant supplements, admission to the intensive care unit and participants’ hesitancy to take part in the study after knowing about the objectives of the study. Out of 64 patients participating in the study, two patients from the intervention group were removed from the study because they were not willing to continue their cooperation and nausea after taking magnesium supplements. Also, 2 people from the control group were withdrawn from this project due to their resistance to further involvement in the study, and the final analysis was performed on 60 people (30 people in each group). Table 1 presents a concise overview of the information about demographic characteristics of the participants involved in this study. The mean (SD) age of the patients was 47.8 (9.50) years and mean weight was 72.47 ± 8.75 kg. Also, 48.34% of participants were male and the rest were female. The participants in the study were from three ethnics: Arabs (21.67%), Fars (21.67%), and Lor/Bakhtiari (31.66%). The mean duration of COVID-19 in magnesium group was 9.90 ± 3.40 days and in control group was 9.10 ± 2.14, without any significant differences (*P* = 0.68). The length of time patients stayed in the hospital was 7.32 ± 5.07 days in the magnesium group and 7.28 ± 4.25 days in the placebo group, and no notable distinction was found between the two groups (*P* = 0.84). Also, in term of Moreover, no substantial disparities were detected between the two groups in term of other baseline characteristics.

**Table 1:** Baseline characteristics of the participants

Variable^1^		Total (*n* = 60)	Magnesium (*n* = 30)	Control (*n* = 30)	p-value
Age (years)		47.8 (9.50)	47.21 (8.47)	48.35 (10.50)	0.78
Height (cm)		163.47 (7.35)	164.25 (8.14)	163.95 (8.15)	0.43
Gender	Male	29 (48.34)	14 (46.66)	17 (56.66)	0.15
	Female	31 (51.66)	16 (53.34)	13 (43.34)	
Weight (kg)		72.47(8.75)	70.68(8.34)	74.38(8.92)	0.43
Ethnic, No (%)					
	Fars	13(21.67%)	7(23.33%)	6(20%)	0.58
	Lor/Bakhtiari	19(31.66%)	9(30%)	10(33.33%)	
	Arab	28(46.67%)	14(46.67)	14(44.67%)	
Duration of COVID-19 (days)		9.52(2.46)	9.90(3.40)	9.10(2.14)	0.74
The duration of having symptoms until positive test (days)		5.62 (2.45)	5.74 (2.68)	5.48 (2.21)	0.68
Acute COVID-19 symptoms, No. (%)					
	Dyspnea	47(78.33%)	24(80%)	23(76.66%)	0.85
	Cough	41(68.33%)	17(56.66%)	24(80%)	0.04
	Sputum	6(10%)	2(6.66%)	4(13.33%)	0.31
	Fatigue	25(41.67%)	15(50%)	10(33.34%)	0.04
	Diarrhea	17(28.33%)	8(26.66%)	9(0.30)	0.51
	Sore throat	30(50%)	14(46.67%)	16(53.32%)	0.31
Duration of hospitalization (days)		7.3(4.10)	7.32(5.07)	7.28(4.65)	0.84
Body temperature		36.68(0.38)	36.60(0.35)	36.76(0.37)	0.73
Lung involvement with COVID-19(%)					
	Mild	23(38.33%)	13(43.34)	10(33.34)	0.31
	Moderate	28(46.67%)	13(43.34)	15(50)	
	Severe	9(15%)	4 (13.32)	5 (16.66)	
Calorie (kcal/day)		1827.34 ± 390.44	1843.18 ± 436.25	1812.52 ± 373.51	0.65
Carbohydrate (%)		54.62 ± 6.19	54.35 ± 6.47	55.83 ± 7.76	0.74
Protein (%)		15.04 ± 3.85	14.73 ± 3.65	15.12 ± 4.12	0.61
Fat (%)		29.43 ± 4.77	29.36 ± 4.53	29.65 ± 5.12	0.55
Vitamin E (mg)		9.36(3.70,15.73)	9.25(3.32,14.32)	10.68(4.48,18.75)	0.43
Vitamin C (mg)		58.19(30.19,103.55)	53.29(32.25,96.76)	63.79(40.69,102.33)	0.22
Vitamin A (mg)		543.27 ± 279.92	537.23 ± 283.93	548.19 ± 265.62	0.63
Magnesium (mg)		360.17 ± 105.90	357.87 ± 110.20	368.32 ± 107.18	0.42

Data are presented as mean (SD) for quantitative and frequency (%) for qualitative variables

Dietary intake of participants showed in Table 1. The mean of calorie intake in magnesium group was 1843.18 ± 436.25 kcal/day and in the control, group was 1812.52 ± 373.51 kcal/day, without any significant differences (*P* = 0.65). There weren’t any significant differences between two groups in term of other macro and micronutrients (*P* > 0.05).

### Effects of magnesium supplementation on serum concentration of magnesium

The average level of magnesium in the blood at the start of the investigation was 2.25 ± 0.28 mg/dl in the intervention group and 2.28 ± 0.37 mg/dl in the placebo group, and there was no substantial variation noticed between the two groups (*P* = 0.55). At the end of the study, we found a significant difference between two groups in term of serum magnesium concentration in the crude (*P* = 0.004) and adjusted model (*P* = 0.001) (Table 2).

**Table 2:** Primary outcomes in a study of the effect magnesium on patients with COVID-19

Variable	Magnesium group		Placebo group			
	Value		Value			P
**Oxygen therapy**						
Need oxygen therapy	9 (30%)		14(46.67)			< 0.001
No need for oxygen therapy	21 (70%)		16(53.33)			

### Primary outcome

The main result of this study was the need to oxygen therapy (Table 3). The results of the present study showed that the number of people who needed oxygen therapy was significantly less in the magnesium group than in the control group (9 patients vs. 14 patients; *P* < 0.001).

**Table 3:** Magnesium concentration at baseline and end of the study

Variable	Magnesium group		Placebo group			
	Value	P^1^	Value	P^1^	P^2^	P^3^
**Magnesium**						
Baseline	2.25 ± 0.28	0.15	2.28 ± 0.37	0.027	0.55	
End of the study	2.34 ± 0.33		2.07 ± 0.32		0.004	
Changes	0.092 ± 0.34		-0.21 ± 0.08		0.003	0.001

P^1^, Paired sample t-test; P^2^, Independent sample t-test, P^3^, ANCOVA adjusted for weight, energy intake

### Secondary outcomes

The average duration of hospital stay did not show any significant difference between the magnesium group and control group (7.32 ± 5.37 days vs. 7.28 ± 4.65 days; *P* = 0.84). In term of oxygen saturation of arterial blood, at the end of the study, we found a significant improvement in the magnesium group than control group (4.55 ± 2.35 vs. 1.8 ± 1.67; *P* < 0.001). However, we couldn’t find any significant differences between two groups in term of respiratory rate (*P* = 0.16), fever (*P* = 0.47), hs-CRP (*P* = 0.65) and TNF-α (*P* = 0.54). There were no notable distinctions observed in the significance of the results after adjusting for confounding variables (Table 4).

**Table 4:** Secondary outcomes in a study of the effect magnesium on patients with COVID-19

Variable	Magnesium group		Placebo group			
	Value	P^1^	Value	P^1^	P^2^	P^3^
**SaO2**						
Baseline	93.13 ± 2.36	< 0.001	94.03 ± 2.26	0.013	0.14	
End of the study	97.68 ± 1.13		95.83 ± 1.36		0.011	
Changes	4.55 ± 2.35		1.8 ± 1.67		< 0.001	0.023
**Respiratory Rate**						
Baseline	21.10 ± 1.81	0.25	19.90 ± 2.00	0.71	0.19	
End of the study	20.26 ± 1.21		19.72 ± 1.38		0.12	
Changes	-0.83 ± 1.31		-0.17 ± 1.55		0.16	0.19
**Fever**						
Baseline	36.60 ± 0.38	0.23	36.76 ± 0.37	0.37	0.35	
End of the study	36.23 ± 0.25		36.39 ± 0.3		0.54	
Changes	-0.37 ± 0.29		-0.37 ± 0.25		0.47	0.52
**hs-CRP**						
Baseline	16.44 ± 3.07	0.009	17.43 ± 2.25	0.017	0.16	
End of the study	14.21 ± 4.31		15.68 ± 3.72		0.17	
Changes	-2.23 ± 4.31		-1.75 ± 3.72		0.65	0.53
**TNF-α**						
Baseline	1.47 ± 4.47	0.16	1.06 ± 1.36	0.283	0.63	
End of the study	0.59 ± 0.47		0.72 ± 1.16		0.55	
Changes	-0.88 ± 1.51		-0.33 ± 1.24		0.54	0.105

P^1^, Paired sample t-test; P^2^, Independent sample t-test, P^3^, ANCOVA adjusted for weight, energy intake and serum magnesium levels at baseline; hs-CRP, High-sensitivity C-reactive protein; TNF-a, Tumor necrosis factor; SaO2, oxygen saturation of arterial blood

In term of mental health related factors and quality of life, we found a significant improvement in the SF-36 MCS score (15.09 ± 11.90 vs. 2.03 ± 10.52; *P* < 0.001) and SF-36 PCS score (16.78 ± 14.32 vs. 3.28 ± 12.35; *P* = 0.01). Also, in relation to the severity of depression, magnesium supplementation caused a significant reduction in the depression score compared to placebo (-5.24 ± 0.76 vs. -1.63 ± 0.68; *P* = 0.03). However, we couldn’t find any significant differences between groups in term of anxiety score (*P* = 104) (Table 5).

**Table 5:** Comparison of mental health related factors between groups at the baseline and end of the study

Variable	Magnesium group		Placebo group			
	Value	P^1^	Value	P^1^	P^2^	P^3^
**SF-36 MCS score**						
Baseline	63.50 ± 16.90	0.003	66.19 ± 19.26	0.109	0.38	
End of the study	78.59 ± 15.73		68.22 ± 17.43		0.03	
Changes	15.09 ± 11.90		2.03 ± 10.52		< 0.001	0.03
**SF-36 PCS score**						
Baseline	78.36 ± 17.45	0.001	80.16 ± 20.15	0.39	0.45	
End of the study	95.14 ± 19.22		83.44 ± 18.67		0.03	
Changes	16.78 ± 14.32		3.28 ± 12.35		0.01	0.02
**BDI total score**						
Baseline	21.98 ± 1.5	0.001	20.75 ± 1.44	0.53	0.87	
End of the study	16.78 ± 1.83		19.12 ± 1.65		0.12	
Changes	-5.24 ± 0.76		-1.63 ± 0.68		0.03	0.03
**State anxiety**						
Baseline	42.15 ± 26.33	0.03	17.43 ± 2.25	0.04	0.31	
End of the study	36.19 ± 27.19		15.68 ± 3.72		0.08	
Changes	-5.96 ± 15.53		-2.92 ± 13.63		0.104	0.126

P1, Paired sample t-test; P2, Independent sample t-test; P3, ANCOVA adjusted for weight, energy intake and serum magnesium levels at baseline; SF-36 MCS, SF-36 mental component score; SF-36 PCS, Physical component score; BDI, Beck’s Depression Inventory

## Discussion

The present study aimed to investigate the effect of magnesium supplementation on clinical and biochemical parameters in patients with COVID-19. The findings of this randomized clinical trial provide valuable insights into the potential benefits of magnesium supplementation in the management of COVID-19 patients. The results of the present study showed that magnesium supplementation among the patients with COVID-19 led to a significant reduction in the need for oxygen therapy, oxygen saturation of arterial blood, quality of life and depression scores. However, we couldn’t find any significant effects on other clinical and biochemical variables.

Magnesium, a vital micronutrient, plays multifaceted roles in cellular metabolism, immune modulation, and pulmonary health [37]. Our study aimed to investigate the impact of magnesium supplementation on both clinical outcomes and biochemical parameters in COVID-19 patients. While the observed improvements in quality of life and mental health may appear to precede changes in hematological factors, it is essential to consider the complex and dynamic nature of magnesium’s effects on physiological processes. The involvement of magnesium in cellular and tissue metabolism is intricate and influenced by multiple factors [26, 38]. Growing evidence indicates that magnesium primarily serves as an important signaling element and metabolite in cellular physiology. Previous findings have emphasized the significance of magnesium in maintaining appropriate immune, vascular, and pulmonary function. In this context, magnesium plays a vital role in the following aspects: ensuring the effective functioning of neutrophils and macrophages, enhancing the cytotoxic activity of T lymphocytes, activating immune cells, and restricting viral replication [27, 39, 40].

Our results demonstrated a significant reduction in the number of patients requiring oxygen therapy in the magnesium supplementation group compared to the control group. This outcome suggests that magnesium supplementation may have a protective effect on respiratory function in COVID-19 patients. These results align with prior research that have highlighted the role of magnesium in maintaining airway smooth muscle tone [41], bronchodilation [42], and reducing airway inflammation [1, 43]. Magnesium’s ability to modulate calcium homeostasis and impact ion channels may contribute to its beneficial effects on respiratory function in COVID-19 patients [18]. As far as we know, in individuals with COVID-19, similar to other viral infections, there may be a higher prevalence of airway hyperreactivity [44]. In relation to this, it is important to consider enhancing ventilation and decreasing airway resistance [45, 46]. Certain medications like β2 agonists and anti-muscarinic agents are employed to alleviate these symptoms. An alternative treatment available for patients who are hospitalized is the use of nebulized magnesium sulfate. Existing literature suggests that magnesium sulfate can induce bronchodilation in individuals with asthma through multiple mechanisms, such as inhibiting calcium influx into the cytosol, preventing acetylcholine secretion, inhibiting histamine release, and enhancing β2 receptor affinity. This, in turn, enhances the bronchodilator effect of β2 agonists [42, 45].

As our understanding of COVID-19’s development has advanced, additional clues shed light on the possible involvement of imbalanced magnesium levels in both the prevention and progression of the disease. Magnesium plays a crucial role in stabilizing the membranes of mastocytes, which are found in the submucosa of the airways and alveolar septa. This function helps prevent their degranulation and the subsequent release of various mediators [28]. Also, this mineral controls the functioning of neutrophils and macrophages by suppressing their priming, inhibiting oxidative burst, and interfering with the Toll-like receptor 4/NFκB axis [47]. In lymphocytes, magnesium regulates the levels of Inositol Triphosphate and diacylglycerol, which are essential second messengers that become active upon the activation of B and T cell receptors. Moreover, magnesium plays a crucial role in defending the body against viral infections, as it is necessary to maintain sufficient levels of intracellular magnesium for the cytotoxic function of T lymphocytes and natural killer (NK) cells [48].

Regarding the secondary outcomes, we observed a significant improvement in oxygen saturation of arterial blood in the magnesium group compared to the control group. This improvement suggests enhanced oxygenation and respiratory efficiency in patients receiving magnesium supplementation. However, we did not observe significant differences between groups in terms of respiratory rate, fever, hs-CRP, and TNF-α levels. These results may indicate that magnesium supplementation primarily benefits respiratory function rather than directly affecting fever or inflammatory markers. In line with our findings, Simental-Mendia et al. in a systematic review and meta-analysis of 11 trials showed that magnesium supplementation hadn’t any significant effect on serum levels of CRP [49]. While our study did not find significant changes in hematological factors such as hs-CRP and TNF-α levels, it is important to acknowledge the multifactorial nature of COVID-19 pathogenesis [50]. The observed clinical improvements may be mediated through alternative pathways, such as magnesium’s neuroprotective properties and its role in neurotransmitter regulation [51]. The reduction in depression scores observed in the magnesium group underscores the potential neuropsychiatric benefits of magnesium supplementation, which may be independent of its effects on inflammatory markers.

In relation to factors impacting quality of life and mental health, our study demonstrated significant improvements in the SF-36 MCS and PCS score in the magnesium group compared to the control group. These findings indicate that magnesium supplementation may have positive effects on mental well-being and overall quality of life in COVID-19 patients. Magnesium’s involvement in neurochemical processes and its neuroprotective properties may contribute to these observed effects [52]. Furthermore, we noted a considerable decrease in depression scores in the magnesium group, indicating a potential role of magnesium in alleviating depressive symptoms in COVID-19 patients. Nevertheless, no notable disparities were detected in anxiety scores between the two groups. In line with our findings, it has been reported in some observational studies that there was an inverse correlation between dietary magnesium intake and risk of depression [53, 54]. Animal studies have definitively demonstrated that magnesium exhibits antidepressant properties. Previous findings indicate that supplementing with magnesium leads to an antidepressant-like effect, reducing the severity of depressive disorders in experimental animal tests and models [55, 56]. Tarleton et al. in a RCT study among the 126 adults showed that magnesium supplementation with daily dose of 248 mg after a duration of 6 weeks, there was a substantial positive improvement in the levels of depression and anxiety [57]. While the link between magnesium and depression is widely acknowledged, the specific mechanism remains uncertain. Nevertheless, magnesium actively participates in various pathways, enzymes, hormones, and neurotransmitters that are intricately involved in the regulation of mood [58]. This compound functions as a calcium antagonist and a voltage-dependent blocker of the N-methyl-D-aspartate (NMDA) channel, which is responsible for controlling the influx of calcium into the nervous system [59]. When magnesium levels are low, an imbalance of calcium and glutamate can disrupt synaptic function, leading to symptoms of depression [43, 60].

The current study has some limitations that should be considered. Firstly, the sample size was relatively small, which might that findings may have limited applicability to a broader context. Conducting larger studies with diverse populations could strengthen the evidence supporting the effects of magnesium supplementation in COVID-19 patients. Secondly, the study duration was relatively short, and the long-term effects of magnesium supplementation on COVID-19 outcomes remain unknown. Studies with longer follow-up periods are needed to assess the sustainability of the observed benefits. Moreover, the study relied on self-reported dietary intake data, which may introduce recall bias and inaccuracies in assessing nutritional status. Future studies could consider using more objective measures of dietary intake, such as food diaries or biomarkers of nutrient status.

## Conclusion

In summary, the findings of this randomized clinical trial suggest that oral magnesium supplementation in COVID-19 patients may have several beneficial effects. Magnesium supplementation was associated with a reduction in the need for oxygen therapy, improved arterial oxygen saturation levels, enhanced mental health-related factors and quality of life, and a decrease in depression scores. However, further research is warranted to confirm these findings, explore the underlying mechanisms, and examine the lasting impacts of magnesium supplementation in larger and more diverse patient populations. The potential benefits of magnesium supplementation in COVID-19 patients make it a promising area for future investigations and could have important implications for clinical management strategies.

## Data Availability

The data that support the findings of this study are available if anyone wants.
